# Appendix

**Published:** 1820-04-01

**Authors:** 


### APPENDIX.

AS the following Discussion relates to physiological points of great importance, and it has appeared in parts, in different publications, and at considerable intervals of time, we have judged it proper to lay the whole before our readers at one view, together with the additional observations relating to it, which have been transmitted to us by Dr. Philip.

In an Appendix to the second edition of Dr. Philip's *Inquiry into the Laws of the Vital Functions*, which was published in 1818, (page 377) he observes,

"The most painful part of my task remains. I do not shrink from it, because the cause of truth, no less than the duty I owe to myself, calls for it. I have been accused of allowing myself to be deceived, or wishing to deceive others, respecting the results of some of my experiments.

"Of four papers which I have had the honour to present to the Royal Society, the two first and the last were ordered to be published. After the paper which was not published had been in the hands of the President for several weeks, for whose obliging conduct towards me on various occasions I must ever feel grateful, I learned, through a channel by which it was impossible for me to be deceived, not only that he had expressed an opinion of it highly gratifying to me, but that he had received a favourable report of it from those, whom he considered best versed in the subject of which it treated, to whose consideration he had submitted it.

"The next intelligence which I received relating to it was, that it was not to be published by the Society; and a few days afterwards I received a letter from a friend, who is in habits of intimacy with many of the members of the Royal Society, in which he states, 'I have good reason to believe, that it has been reported in and out of the Royal Society, that your observations and conclusions are not correct, and that the stomach cannot be made to act by the process you have decribed." I had soon reason to believe, from several other circumstances, that the President and others had changed their opinion respecting the merits of my paper.

"At this time, I viewed these circumstances with surprise. They appeared to me inexplicable, for I could not conceive the possibility of what the following incident disclosed. One thing alone I was assured of, that such men as those who had, in the first instance, given an opinion respecting my paper, would not change it on slight grounds.

"As the paper had been written from detached notes, and I had kept no regular copy of it, I wrote to one of the Vice-Presidents of the Society, requesting, that as it was not to be published, it might be returned; or, if that could not be done, that a copy of it might be sent to me. The Vice-President's letter is now before me, in which he says, —'I applied to the council for a copy of your paper, and gave directions to the clerk to copy it.' In addition to the copy of my paper, thus made out by order of the council, which also now lies before me, I found, to my great astonishment, written in the same hand with the copy of the paper, the following observation of the clerk, with the subjoined account of an experiment.'

'N. B. The following is a copy of a paper, written in a different hand, and pinned in page 13, over part of the account of Experiment 3,* without any reference to the text whatever.'

'Two rabbits, which had had no food for seventeen hours, were allowed to eat parsley. The nerves of the were then divided in the neck of each. One of them was allowed to remain quiet. A slip of tin-foil was connected to the lower divided ends of the nerves of the other rabbit, and another piece of tin-foil, an inch square, was applied to the abdominal muscles over the stomach, and under the integuments, by means of a wound in the latter. The tin-foil over the stomach was connected with a wire communicating with one end of a voltaic battery of twenty plates, and occasional contacts were made (about three or four times a minute) between a wire connected with the other end of the battery and the tin-foil in the neck. The influence of the battery was sufficiently strong to excite slight contractions of the muscles of the fore legs. This process was continued during five hours, at the end of which period both rabbits were killed.

'* The first of my galvanic experiments.'

'On examining the stomach of the animal, which had been subjected to the influence of the battery, it was found much distended with food, the parsley was principally in the cardiac portion, and near the œsophagus it appeared to have undergone no alteration; and below this it was mixed with the other food in the stomach, so that no accurate observation could be made on it.

'The stomach of the other rabbit was examined by the side of the first, so that they might be compared together, and the appearances were precisely the same with those which have been just described. The contraction in the centre of the stomach was somewhat greater in the galvanised stomach than in the other.'

"The above addition to the copy of my paper, sufficiently explained the circumstances attending its presentation. An account of an experiment, which, on a superficial view, or to those whose attention had not been particularly directed to the subject, appeared to be a repetition of my galvanic experiments, affording a result in direct opposition to them, was laid up in the archives of the Royal Society; and, without any thing having been said to me on the subject, pinned to the account of my experiments; from which it would appear, that I had either been deceived in their result, or wished to deceive others. I had either been in the most unaccountable degree careless or uncandid.

"How far the experiment just laid before the reader warrants this conclusion, a very short comparison of it with my galvanic experiments will shew. The inference afforded by them, that when the eighth pair of nerves is divided, and the digestive power of the stomach thus wholly suspended,* it may be renewed and supported by passing a continued stream of galvanism through the stomach.† Let us inquire whether the experiment in question interferes with this inference. In repeating my experiment, the first step, of course, is to divide the eighth pair of nerves. Without this the animal is not in the state which affords an opportunity of making the experiment at all. If the nervous influence which Nature supplies is not withdrawn from the stomach, no addition made to it can be of any avail in promoting the process of digestion. This

"* The muscular power of the stomach remains after the division of these nerves." See page 154.

"† My experiments also relate to the lungs, but the author of the above experiment confines his attention to the stomach."

organ already possessing as much nervous influence as the fluids supplied to it require, can make use of no more. The first question, then, with respect to the above experiment is,—Were the nerves of the eighth pair divided previously to the application of the galvanism? *They were not.* The author does not pretend to say that they were. He leaves a blank for the name of the nerves divided. He mentions none of the symptoms which uniformly follow the division of the eighth pair of nerves; and the state of the contents of the stomach, which he describes, is such as it is never found to be, after the animal has survived the division of these nerves for five hours.* The remaining question is,—Was a continued stream of galvanism sent through the stomach?† *It was not*, for the author of the experiment states, that only 'occasional contacts were made (about three or four times a minute) between a wire connected with the other end of the battery and the tin-foil in the neck.' *In what essential respect then does this experiment resemble my galvanic experiments?*‡

"My first impulse on the receipt of the copy of my paper, was to address a Letter publicly to the President of the Royal Society, requesting an explanation of the above most extraordinary occurrence. On more mature reflection, however, and by the advice of a distinguished Fellow of the Royal Society, to whom I had mentioned all the circumstances, and my intention of publicly addressing the President, I resolved to adopt a less painful, though more tedious means of securing to my experiments the credit to which I knew them to be entitled. I therefore merely published, above a year afterwards, in an Appendix to this Inquiry, the account of the experiment which I had received, without saying from what quarter I had received it, pointing out that it did not bear on the subject in question. I was convinced that its author, of whom I am to this moment ignorant, would see or hear of

" * See pages 154 and 155 of this Treatise. The contents of the stomachs of both rabbits were in a perfectly healthy state. The author of the experiment was deceived in supposing any part of the old and new food mixed together, by a circumstance explained above." See pages 142 and 143.

" † I cannot help remarking, that the introduction of the tin-foil under the skin, perhaps the most painful part of the experiment, is an instance of useless cruelty, the skin being a sufficiently good conductor of galvanism."

" ‡ I have not thought it necessary to mention several deviations from my experiments of less importance, none of which, however, were allowable."

this notice of it; and hoped, that if he could not bring himself openly to acknowledge the irrelevancy of his experiment, he would at least take some step to do away the impression it had made. It is now, however, nearly a year since the first edition of my Inquiry appeared; and I have means of knowing that no such step has been taken. I therefore recur to my original intention, and thus publicly address the President and Council of the Royal Society; satisfied from the proofs of their candour which I have in other instances received, that they will do what the cause of truth requires from them—that they will call on the author of the above experiment, either to shew in what respect it opposes the result of my experiments, or publicly to confess his error."

This appeal remained unnoticed till April 1819, when the following paper appeared in the Quarterly Journal of the Royal Institution.

" Art. XXI. *A few Facts relative to* Dr. Wilson Philip's *Attack on the President and Council of the Royal Society*.

" Dr. Wilson Philip, in the 279th and following pages of the second edition of his Experimental Inquiry into the Laws of the Vital Functions, has made an attack upon the President and Council of the Royal Society, for having had a paper pinned into the copy of his experiments and observations, which is preserved in their archives; and desires to know how it came there. The Editor of this Journal hopes the explanation that follows will prove satisfactory, and exculpate the Council from any intention of hostility towards Dr. Wilson Philip.

" When the paper was read before the Society, there were many members who thought it was right that one of the experiments should be repeated. Three members of the society undertook this task, one conducting the galvanic part; another, the anatomical part; and the third, who was not made acquainted which was the galvanized rabbit, was called in after the experiment was over, to decide upon the stomach in which the food was most acted upon: that the experiment might be repeated with the greater accuracy, the paper was put into these gentlemen's hands, who implicitly followed the directions contained in it. These gentlemen were quite competent to the task, and each cofined himself to his own department; the third, who was employed to examine the contents of the two stomachs, after an accurate inspection, was unable to detect the slightest difference between them. This result was stated to the President, to whom it was also explained, that the rabbit, which is a species of ruminant, does not digest its food till it has gone through a previous process of maceration, and is therefore not so well fitted for such experiments, as animals that live on animal food. The manuscript dissertation of Dr. Philip was then returned to the clerk of the society; and a minute, made during the time of the experiments, was accidentally left in it.

"Dr. W. Philip's account of his experiment, and that of the experiment made by the members of the Royal Society, are subjoined, for the information of the public.

"*Experiment made by Dr. Philip.*—The hair was shaved off the skin over the stomach of a young rabbit, and a shilling bound upon it. The eighth pair of nerves were then divided, and about a quarter of an inch of the lower part of each coated with tin-foil. The tin-foil and the shilling were connected with the opposite ends of a galvanic trough, containing forty-seven four-inch plates of zinc and copper, the intervals being filled with muriatic acid and water, in the proportion of one of acid to seven of water. The galvanic influence produced strong contraction of the muscles, particularly of the fore limbs, and frequently the pain it occasioned was such that the animal cried out violently, and made it necessary for a little to discontinue the process.

"For five hours the animal continued quite free from the symptoms which follow the division of the eighth pair of nerves in rabbits. It had neither vomited nor been distressed with dyspnœa. It had not eaten any thing after the nerves were divided. At this time the power of the trough became much weaker, so that it produced no visible effect on the muscles. The respiration now began to be disordered. In a quarter of an hour it became so difficult, that the animal appeared to be dying. It was gasping. Acid was put into the trough till the galvanic power became as great as at first. Soon after this, the animal ceased to gasp, and breathed with much greater freedom. The galvanic process was several times discontinued and renewed, so that we repeatedly saw the gasping and extreme dyspnœa return on discontinuing, and disappear on renewing it. The animal seemed now much exhausted, and could scarcely raise itself. It had been held down on its side during the whole experiment. It died in six hours after the division of the nerves.

"On opening it, we found the œsophagus perfectly natural, and no food in it. The stomach was not larger than usual. The food had undergone considerable change. The appearance and smell of the parsley were gone. The smell was that of the rabbit's stomach while digestion is going on, which is peculiar. Mr. Hastings, who has been much accustomed to examine the stomach of rabbits under various circumstances, said that digestion was nearly as perfect as it would have been in the same time in a healthy rabbit.

" The membrane of the trachea was of its natural colour, and there was no fluid in it. The ramifications of the bronchiæ in the left lung were quite free from frothy mucus. There was some fluid in the right lung, though it did not appear much gorged; there was one dark spot on it. The lungs collapsed imperfectly on opening the chest.

" This rabbit had not eaten any thing for twelve hours till within three hours of the operation, during which it was allowed to eat as much parsley as it chose."

" *Experiments made by Three Members of the Royal Society.*

" Two rabbits, which had had no food for seventeen hours, were allowed to eat as much parsley as they chose. The nerves of the par vagum were then divided in the neck of both rabbits. One of them was allowed to remain quiet. A slip of tin-foil was connected to the lower divided ends of the nerves of the other rabbit, and another piece of tin foil, an inch square, was applied to the abdominal muscles over the stomach and under the integuments, by means of a wound in the latter. The tin-foil over the stomach was connected with a wire, communicating with one end of a charged voltaic battery of twenty plates, and occasional contacts were made (about three or four times in a minute) between a wire connected at the other end of the battery, and the tin-foil round the nerves in the neck. The influence of the battery was sufficiently strong to excite slight contractions of the muscles of the fore legs. This process was continued during five hours, at the end of which time both rabbits were killed. On examining the stomach of the animal, which had been subjected to the influence of the battery, it was found much distended with food. The parsley was principally in the cardiac portion: near the œsophagus it appeared to have undergone no alteration, and below this it was mixed with other food in the stomach, so that no accurate observation could be made on it.

" The stomach of the other rabbit was examined by the side of the first, so that they might be compared together, *and the appearances were precisely the same as those which have been just described.* The contraction of the centre was somewhat greater in the galvanised stomach than in the other*.

" Dr. Philip says, that in his experiment there were muscular contractions produced by the galvanic influence; which proves that he employed it, not in a continued stream, but by occasional contacts, as in the experiment made by the members of the Royal Society.

" Dr. Philip, in his account of the latter experiment, has left a blank where the names of the nerves should have been inserted, whereas the words *par vagum* were written as plain as any other part of the paper.

" In this experiment, it was not observed that the galvanic influence produced any change on the respiration, the dyspnœa being equal, whether the battery was in force or not: In Dr. Philip's first experiment, he says, that the dyspnœa was entirely relieved by the galvanism; yet the animal died at the end of six hours. What, then, was the cause of death?

" The gentlemen concerned in the experiment, not being able to devote so much time as in Dr. Philip's second experiment, the experiment was continued during five hours, which is nearly the time with that in Dr. Philip's first experiment, which was continued for six hours. One of the gentlemen employed in making these experiments, started many objections to dividing the par vagum in the neck; he stated, that if the nerves of the eighth pair be divided in the neck, the digestion of the animal is impaired, but this affords no satisfactory proof of these nerves exercising a direct influence over the functions of the stomach, since these functions may be affected, in consequence of the disturbed state of the respiration, which this injury occasions, and of the imperfect alteration of the blood in the lungs. The following experiments, in which the same nerves were divided below the origin of the branches, which are distributed to the lungs, appeared to him to be free from objection, and are here given with a view to throw further light on this subject.

" *Experiment* 2.—In a young cat, the termination of the nerves of the eighth pair, on the cardia of the stomach,

* " In this experiment, the respiration was affected, as usual, after the division of the eighth pair of nerves, and it was not observed that the dyspnœa was at all relieved by the galvanic influence.

were carefully divided. The animal was perfectly well afterwards; was lively; ate his food as usual; and the respiration was, of course, unaffected. At the end of a week, and three hours after having been fed with meat, the cat was killed.

"On dissection, digestion was found to have been going on as usual. The food in the stomach was in a great measure dissolved, and the thoracic duct and lacteals were distended with chyle, having the ordinary appearance. The nerves were carefully traced, and it was ascertained that not the smallest filament had been left undivided. This experiment was repeated with exactly similar results.

"These experiments appear to set this inquiry at rest, and to disprove the experiments made by Dr. Wilson Philip. It was intended to have laid them before the Royal Society, but the morbid sensibility shown by so many members on hearing the experiments detailed by Dr. Wilson Philip, deterred the author from running the risk of so soon again awakening these feelings."

To the above Paper Dr. W. Philip made the following Reply in the next number of the same Journal.

"Art. XVIII. Dr. Wilson Philip's *Reply to some Observations relating to his Inquiry into the Laws of the Vital Functions in the last Number of the Quarterly Journal, in a Letter addressed to* W. T. Brande, Esq.

"*Worcester, May* 20, 1819.

"Sir,

"As you have in the last number of the *Quarterly Journal*, which did not fall into my hands until the day before yesterday, inserted a paper relating to some experiments of mine, in which there appears to me to be several mis-statements, you will, I am persuaded, do me the justice to insert the following observations in the next number of that work. Why you should say, that in the Appendix to the second edition of my *Inquiry into the Laws of the Vital Functions*, I have made an attack on the President and Council of the Royal Society, I am wholly at a loss to understand; for I cannot conceive, Sir, that you are actuated by a wish to prepossess the feelings of the reader against me, before you make an appeal to his judgment; nor can I, on the other hand, see any thing in my Treatise which can possibly be construed into an attack on the President and Council of the Royal Society, for whom I have ever felt and expressed the greatest respect; and whose conduct towards me has been calculated to excite no sentiments but those of esteem and gratitude, as I have hinted in more than one passage of the Appendix to which you allude.

"I had found, from various circumstances there stated, that an implied charge had been brought against me in the Royal Society. I only requested that the President and Council would call on the author of that charge, either to substantiate what he had advanced, or acknowledge his error. A regard for my character required this step, and any person who will take the trouble to read the Appendix in question, will admit that I did not take it hastily.

"Permit me, in the first place, to mention two inaccuracies, which it appears, from the observations in the *Quarterly Journal*, the clerk of the Royal Society must have committed in the account he sent to me of the experiment supposed to invalidate the result of mine. In that Journal it is said, 'Dr. Philip, in his account of the latter experiment, has left a blank, where the name of the nerves should have been inserted; whereas the words *par vagum*, were written as plain as any other part of the paper.' In the copy sent me, *par vagum* is not mentioned, but a blank left, as any gentleman may satisfy himself by requesting a friend here to inspect it. Mr. Andrew Knight, a distinguished member of the Royal Society, has seen it. In the *Quarterly Journal* it is said, 'The manuscript dissertation of Dr. Philip was then returned to the clerk of the Society, and a minute made during the time of the experiments *was accidentally left in it*.' In the clerk's account it is stated, 'The following is a copy of a paper written in a different hand, and *pinned in page* 13, *over part of the account of Experiment* 3.'

"Of the experiment, an account of which was either accidentally left in, or pinned to the corresponding experiment in my paper, the observations in the *Quarterly Journal* give the following account. 'When the paper was read before the Society, there were many members who thought it was right that one of the experiments should be repeated. Three of the members of this Society undertook this task, one conducting the galvanic part, another the anatomical part, and the third, who was not made acquainted which was the galvanized rabit, was called in after the experiment was over, to decide upon the stomach in which the food was most acted upon. That the experiment might be repeated with the greater accuracy, the paper was put into these gentlemen's hands, who implicitly followed the directions contained in it.' This is what I

have denied, because, for example, in my experiment a continued stream of galvanism was maintained, while in that here alluded to, only occasional contacts were made between a wire connected with the other end of the battery and the tin-foil in the neck. But it is stated in the *Quarterly Journal*, that 'Dr. Philip says that in his experiment there were muscular contractions produced by the galvanic influence, which proves that he employed it not in a continued stream, but by occasional contacts, as in the experiment made by the members of the Royal Society.' The gentlemen who make this statement must, indeed, be unacquainted with the effects of galvanism on the living animal body, when they suppose that a continued stream of galvanism of a certain power, applied as in my experiments, does not occasion a constant repetition of contractions in the neighbouring muscles. Not only the continued stream, but the requisite power of galvanism, was wanting in their experiment; because it requires a much greater power to occasion repeated contractions of the muscles by a continued stream, than by occasional contacts of the metals. The above gentlemen, therefore, so far from repeating the experiment in the way in which I made it, here deviated from it in so important a circumstance, that had their experiment in other respects resembled mine, this deviation alone must necessarily have occasioned a different result.

"The observations in the *Quarterly Journal* thus proceed. 'These gentlemen were quite competent to the task, and each confined himself to his own department; the third, who was employed to examine the contents of the stomachs, after an accurate inspection, was unable to detect the slightest difference between them. This result was stated to the President, to whom it was also explained, that the rabbit, which is a species of ruminant, does not digest its food till it has gone through a previous process of maceration, and is therefore not so well fitted for such experiments as animals that live on animal food' I have, with the assistance of Dr. Hastings and Mr. Sheppard, physician and surgeon to the Worcester Infirmary, examined, by killing the animal at various periods of digestion, the stomach of about a hundred and thirty rabbits, and they will attest the accuracy of what I say, when I declare that as far as we could judge from this extensive set of experiments, the results of which are stated at length in my *Inquiry*, the information thus given to the President of the

Royal Society is incorrect. The rabbit is, in no sense of the word, a species of ruminant.*

"In stating the result of their experiment, the above gentlemen observe, 'On examining the stomach of the animal which had been subjected to the influence of the battery, it was found much distended with food. The parsley was principally in the cardiac portion. Near the œsophagus it appeared to have undergone no alteration, and below this it was mixed with other food in the stomach, so that no accurate observation could be made on it.' In this experiment the *par vagum, it is said, had been divided in the neck*, and the animals had survived its division five hours, having ate as much parsley as they chose immediately before the operation, and after having fasted for seventeen hours; I may here, in the first place, observe, that the gentlemen, in this part of their account, again shew how little they are acquainted with the subject in discussion. In the stomach of the rabbit the old and new food are never mixed†. In the healthy state of the stomach, the old and new food may, indeed, to a superficial view, appear to be mixed, for a reason pointed out in the 143d page of my *Inquiry*; but the appearance which leads to this misconception is never observed under the circumstances of the above experiment, which I have many times witnessed. I subjoin the following declaration of Dr. Hastings.

'I hereby declare, that I have more than twenty times had occasion to perform the experiment of dividing the *par vagum* in the neck of the rabbit, when it had been allowed to eat parsley after a fast of many hours, and examined the contents of the stomach, the animal having been allowed to live for five or more hours after the operation, and I never found them in the state described in the *Quarterly Journal*, nor in any other state, but that described in the 154th page of Dr. Philip's *Inquiry into the Laws of the Vital Functions*.

(Signed) 'CHARLES HASTINGS,

'*Physician to the Worcester Infirmary*.'

"My opponents in the *Quarterly Journal* forget to state, that in the most conclusive galvanic experiment on rabbits

---

* It would have been candid in the gentlemen to have stated here, that an account of this experiment on a carnivorous animal, the dog, performed with the same result in the presence of several medical gentlemen, is given in my *Inquiry*.

† See the 142 page of the second edition of my *Inquiry*.

related in my Treatise, the galvanism was applied for *sixteen hours. They did not allow themselves time to apply it even as long as in that, in which its effect from the short time of its application, six hours, was acknowledged to be imperfect.* The galvanised animal was never voluntarily killed by me. On the contrary, its life was always prolonged as much as possible. The galvanism, we have seen above, was neither applied in the same way, nor of the same power, as in my experiments. With what propriety, then, to say nothing of less important deviations, can these gentlemen, even according to their own account, maintain that they *implicitly followed the directions given in my paper?*

"It is observed in my Appendix, that no notice is taken of the symptoms which follow the division of the *par vagum* in the account of their experiment. In that they now publish, indeed, they state that dyspnœa occurred*; but still omit to mention the ineffectual attempts to vomit, which as constantly follow the division of the *par vagum*, as the dyspnœa does.

"For what purpose the experiment, marked Experiment II, in the *Quarterly Journal*, is detailed, I know not; as in all my experiments the nerves were divided in the neck, where, it has long been known to physiologists, their division destroys the power of the stomach.

"I am asked, of what the animal died, in six hours, in my experiment quoted in the above Journal, if not of dyspnœa. The reader will find this question answered in the account of Experiment 71 of my *Inquiry*.†

"In the latter part of the observations in the *Quarterly Journal*, the authors allude to what they term 'The morbid sensibility' of many of the members of the Royal Society, to some of my experiments which were made on living animals; but this sensibility can neither be termed morbid nor unreasonable, if, as I was informed, reports were industriously circulated, that these experiments were

---

* "They observe in a note—'In this experiment the respiration was affected as usual after the division of the eighth pair of nerves, and it was not observed that the dyspnœa was at all relieved by the galvanic influence.' In reply to this I can only observe, that Dr. Hastings, Mr. Sheppard myself, and several others, have very frequently witnessed the application of galvanism in the dyspnœa occasioned by dividing the *par vagum*, and never saw it fail to relieve this symptom, when it was applied of the proper strength. It is remarkable that so striking a difference of result should not have been mentioned in the first account of their experiment."

† "When I refer to my *Inquiry*, the number of the page or experiment is always that of the second edition."

not only useless, but that from an erroneous choice of the the animal on which they were made, it was impossible they should have been otherwise.

"I have now stated the reasons which, I believe, entitle me to say, that the report of the three gentlemen in the *Quarterly Journal*, leaves the statement, in my Appendix, exactly where they found it; and indicates a degree of information which but ill accords with the confident style in which they write.

"I cannot conclude without adverting to the circumstance of these gentlemen still persisting to conceal their names. This is seldom done on such occasions without a strong motive. I hope theirs is not such as the line of conduct they have for several years pursued, cannot fail to suggest. That three members of so respectable a Society, forgetful of better feelings, the boast of men of science, should combine to depreciate the exertions of an individual, is what I shall be very slow to believe.

"I have the honour to be,
"Sir,
"Your very obedient humble servant,
"A. P. W. PHILIP."

No reply was made by the three members of the Royal Society to the above observations of Dr. W. Philip; and here the discussion rested till the appearance of Dr. Cooke's work on Nervous Diseases, in the beginning of the present year. In consequence of some observations in the introduction to this work, a correspondence took place between Dr. Philip and Mr. Brodie, which, with some additional observations, was published by the former gentleman, in two of the Medical Journals of last month, under the following title.

"Dr. Philip's *Reply to* Mr. Brodie, Dr. C. H. Parry, *and Others.*

"Dr. Cooke, in the Introduction to his work on Nervous Diseases, relates several experiments performed by Mr. Brodie, the results of which, Mr. Brodie thinks inconsistent with certain inferences in my Inquiry into the Laws of the Vital Functions. Dr. Cooke also quotes, from the Quarterly Journal for April last, the observations of three members of the Royal Society, who think that I was deceived in the result of certain experiments related in the above Inquiry; but without noticing my reply to these observations in the following number of the same

Journal. I addressed a letter to Dr. Cooke, complaining of this circumstance, to which he returned an obliging answer, with permission to make it public. He says:—'The Quarterly Journal for April last was sent to me by a friend, who thought that the experiments related in it were connected with the subject of my introduction. I have seen no other number of that Journal, and was not aware that you had made a reply to the observations contained in it, otherwise I should have thought it a duty to have detailed that reply. If my book should go to a second edition, which I think not improbable, I shall be happy to supply the deficiencies of the first.'

"The introduction to Dr. Cooke's work also induced me to address the following letter to Mr. Brodie:—

"*Worcester, January* 25, 1820.

"Sir,—A gentleman from London informed me, that he heard it publicly mentioned, that you are one of the gentlemen who gave the statement in the Quarterly Journal of April last, in answer to some observations in the Appendix to the second edition of my Inquiry into the Laws of the Vital Functions. This report appears to be confirmed by what is said in the 129th and following pages of Dr. Cooke's late Treatise on Nervous Diseases. It is with considerable surprise, that I have there seen the experiment, which was detailed in the above-mentioned statement in the Quarterly Journal, again brought forward as affording a refutation of the results of some of my experiments, after I have repeatedly pointed out, that the circumstances of that experiment are *in no essential respect similar* to those of the experiments in question; and it is universally admitted, that in such experiments, even *the slightest deviation* may influence the result. The only thing which can excuse Dr. Cooke's quoting from the Quarterly Journal the experiments and observations of my opponents, and wholly passing over in silence my reply to them, is, that he may not have seen the following number of that Journal, which contains my reply.

"I ask you, Sir, as a gentleman, and a man of science, to say, whether the above mentioned experiment can be regarded as a fair repetition of my galvanic experiments?

"I am mortified to find, that I have made myself so ill understood by you in my Inquiry into the Laws of the Vital Functions, that you have, in Dr. Cooke's work, brought forward several experiments, with a view to refute certain parts of that Inquiry, which do not, as far as

I can judge, at all interfere with any opinions maintained in it.

"In the first of these experiments, the nerves sent by the eighth pair to the cardiac portion of the stomach, were divided, and at the end of a week and three days, digestion was found to be going on as usual. This experiment was related to me by Sir Everard Home in a letter, which I still have by me, as performed by Magendie, more than a year before the publication of the first edition of my Inquiry, in the 168th page of which it is mentioned; and I cannot perceive any reason why a different result should have been expected from it. It is shown, in my Inquiry, that if any considerable part of the influence of the brain or spinal marrow be withdrawn from the stomach and lungs, the secretions of these organs are deranged. The par vagum evidently conveys to the great chain of ganglians the influence of the brain. When it is divided at a short distance from its origin, the influence it conveys is cut off; but I cannot perceive how this can at all be done by dividing the particular branch of this nerve which goes to the cardiac portion of the stomach. Before it arrives at this place, it has formed innumerable connexions with the great sympathetic and ganglians. They have received from it the influence of the brain; and if nerves going to any particular organ be divided, there are every where, such are the precautions of Nature, numerous anastomosing branches still capable of conveying the necessary influence, as long as it is duly supplied from its sources.

"Had you, Sir, been more conversant with the effects of dividing the par vagum, you would not, I think, have made the observations, quoted in Dr. Cooke's work, relating to the influence of the state of the lungs on the stomach, after the division of these nerves in the neck. The dyspnœa is at first so slight, as often to be hardly perceptible; sometimes, indeed, it is not at all so. It only becomes considerable as phlegm accumulates in the lungs; yet, if the animal be allowed to eat as soon as the nerves are divided, efforts to vomit immediately ensue. In one case, in which Dr. Hastings had divided them, and the animal was immediately allowed to eat, the food it had taken, in consequence of the efforts to vomit, accidentally got into the trachea in such quantity as to occasion suffocation, and the animal died within five minutes after the division of the nerves, and without any dyspnœa, previous to the act of suffocation, having been observed. If you will take the trouble to re-peruse the account of my galvanic experiments, you will find, that the digestion was sometimes most impaired when the dyspnœa was least so, and *vice versa.*

"The observations just made, respecting the division of the cardiac nerves, apply, if possible, with greater force to the experiments in which the great nerves of the leg were divided. Dr. Monro, I believe, relates similar experiments in his lectures, but without drawing your inferences from them. It is well known, that nerves accompany the vessels throughout every part of the body in such number, that could we destroy all other parts of a limb, leaving the nerves alone, they would still give the appearance of the entire limb. Now, it is shown, by experiments related in the above Inquiry, that the nerves which supply the vessels belong to the ganglian system; an inference well supported by the observations of the Anatomist; and, that it is on these nerves, and not on the nerves immediately derived from the brain and spinal marrow, that secretion depends. The use of the nerves you divided, is to convey impressions to and from the limb, and to excite the muscles of voluntary motion. It is true, they receive twigs from the ganglian nerves, as all parts do; but the division of these can be of little consequence, as the number of ganglian nerves and their anastomoses may be said to be almost without end. It is the character of the ganglian nerves, that they every where anatomose, and that each may convey the influence of many, while the nerves, arising directly from the brain and spinal marrow, convey the influence only of that part of those organs from which they take their rise. These facts are illustrated by many experiments related in my Inquiry. It might as well be said, that the blood supplied by any of the great arteries of a limb, is not essential to the health of the limb, because, when the artery is secured by ligature, the anastomosing branches can perform its functions; as that the division of any ganglian nerve, not impairing the function of secretion, proves that the influence it conveyed is not necessary to the due performance of that function. While the source of nervous influence is entire, Nature has provided many channels for that part of it on which life depends.

"The next experiment related in Dr Cooke's work, is one in which the source of nervous influence was lessened, and there were evident marks of deranged secretion in the wound. It is true, that you observe, 'the circumstance of the union being incomplete may be reasonably attributed to the animal being in a torpid state, and remaining apparently without nourishment for many months.' But all who have been much in the practice of experimenting on frogs, know that their health is not readily influenced by causes which greatly impair that of warm-blooded animals. Granting, however, that the causes you state did, to a certain degree, operate, will it be admitted, that in estimating those which prevented the proper healing of the wound, nothing is to be ascribed to such a privation as that of the lower part of the spinal marrow? The effect was such as corresponds with the results of my experiments. Even in those in which the greatest part of the spinal marrow was destroyed, which could be done without immediately destroying life, the stomach was not more affected than by dividing one of the eighth pair of nerves; and when the whole of the lumbar portion was destroyed, some degree of digestion still went on, even in the warm-blooded animal, which is so much less tenacious of the living powers than that of cold blood. It appears from many experiments of M. Le Gallois, as well as of my own, that the lower part of the spinal marrow is the least essential of those parts which prepare the nervous influence.

"The inference to which your view of the experiments, which Dr. Cooke quotes from you, have led you, that only some of the secretions are under the influence of the nervous power, (Dr. Cooke's work, page 134,) seems unphilosophical, and would, therefore, on this account alone, have appeared highly improbable.

"When I consider the nature of this letter, Sir, I need not, I trust, beg of you to favour me with as early a reply to it as your engagements will admit of.

"I have the honour to be,

"Sir,

"Your very obedient humble servant,

(Signed) "A. P. W. PHILIP."

"The following is the answer which I received from Mr. Brodie.

"16, *Saville Row, February* 4, 1820.

"Sir,—You were correctly informed, that I was one of the three Fellows of the Royal Society who were formerly requested to undertake the repetition of your experiment on the application of the galvanic influence to the stomach, after the division of the nerves of the eighth pair. After reading your observations on our experiment, I can discover only one point of difference between it and the original experiment made by yourself, namely, that in ours the galvanic influence was applied by a succession of contacts instead of a continued stream. How far this may have affected the results I will not pretend to determine: but as you suppose that it might have done so, I have (since I had the honour of receiving your letter of the 25th ult.) made the experiment again, taking care that it should very accurately resemble yours in this and in all other respects. The rabbit was fed with parsley; and immediately after the nerves of the eighth pair were divided, he was subjected to the influence of the galvanic battery, and this was continued for upwards of seven hours; at the end of which time the contents of the stomach were examined, and found to have precisely the same characters, the same odour, appearance, and consistence, as in another rabbit, which had been fed in the same manner, and in which the nerves had been divided at the same time, but on which the galvanic battery had not been employed.

"You may be assured, that neither my friends nor myself have the smallest desire to depreciate the value of your labours; and I hope that I have sufficient zeal in the cause of science, to have experienced a real pleasure, if I could have met with results similar to those obtained by yourself. That I have not done so, is not to be considered as my fault, and ought not to be regarded by you as a matter of offence. If the subject be worthy of further investigation, I doubt not that some competent experimenter will, sooner or later, undertake to prosecute it: for myself, I take the liberty of declining to enter into any controversial discussion, for which I have neither leisure nor inclination.

"I beg to offer to you my apologies for not having acknowledged your letter sooner; the delay has arisen from my desire to make a second repetition of your experiment previous to my writing to you.

"I have the honour to be,

"Sir,

"Your obedient servant,

"B. C. BRODIE.

"To Dr. A. P. W. Philip, Worcester."

"In reply to Mr. Brodie's letter, I wrote the following, enclosing the declarations of Dr. Hastings, and Mr. Sheppard, which I am about to lay before the reader.—

"*Worcester, February* 7, 1820.

"Sir,—I was yesterday favoured with your reply to my last. You will be troubled with another letter, which I

transmitted through an uncle, who resides in London, and who left Worcester before your letter reached me. I hope you will believe, that it is impossible for me to associate any degree of unfairness with the opinion I have always entertained of you; but the experiment in question has been so often repeated, and with such circumspection, and that by different people, that there are no physiological facts of which I am better assured, than that a certain power of galvanism will remove dyspnœa, and restore to the stomach the power of altering the food after the eighth pair of nerves are divided in the neck. One man may be deceived; but that the number who have witnessed this experiment, and that repeatedly, should have been so, is impossible. I am therefore persuaded that the circumstances of your experiment and mine have differed in some essential respects, although you are not at present aware of it.

"I can form no judgment either of the circumstances of your experiment, or the precise result, till you have answered the following questions, which you will particularly oblige me if you will have the goodness to do on receipt of this letter.

"1. Was the animal fed, after a fast of some continuance, immediately before the experiment?

"2. Was the twitching of the fore-legs constant during your experiment?

"3. Was the dyspnœa, occasioned by the division of the nerves, relieved by the galvanism?

"4. Did vomiting come on in the galvanized rabbit?

"5. What was the appearance of the food in the stomachs, as exactly as it can be stated?

"You do not, I hope, Sir, think me so illiberal, not to say irrational, as to be offended at any one for observing different results from any of my experiments; but you will allow, that repeating an experiment made by me, without due attention to what I consider its most essential circumstances, and laying the repetition before the Royal Society, and thus influencing many of its members against me, without the subject having been mentioned to me, and, consequently, without an opportunity of vindicating myself having been given to me, are just causes of complaint.*

---

* "I might also have stated, in the above letter, that the circumstance of the names of all the gentlemen who made the experiment having, till now, a space of several years, remained unknown to me, greatly increased the difficulty of proving the truth of my experiment."

" I can truly say, I believe that I have as little leisure or inclination for controversial discussion as you have; but in one particular I differ from you. It is certainly optional with every man, whether he will enter into discussions or not; but when he may retire from them, is not equally so. I can feel no wish to retire from the discussion in question till it is finally settled; and if we cannot otherwise discover the circumstance which has caused so great a difference in the result of our experiments, I shall be happy (as I shall have occasion to be in London next autumn, if not sooner,) to repeat my experiment in your presence, and that of some other of the most eminent men of our profession; and their report will determine the question.

" I send, on the other side of this paper, the declarations of Dr. Hastings, aud Mr. Sheppard, both men of the highest respectability; the former well known to Dr. Burrows, Dr. James Johnson, and others of our profession in London; the other to Mr. Cline, Mr. Astley Cooper, and others.

" I have the honour to be,
" Sir,
" Your faithful humble servant,
(Signed) A. P. W. PHILIP.*

To B. C. Brodie, Esq.

" The enclosed declarations.—

" I hereby declare, that having very often divided the eighth pair of nerves in the neck of the rabbit, and always found that it immediately put a stop to digestion, the food eaten previously to the division of the nerves being found in the stomach unchanged, however long the animal lived after the division of the nerves, I was requested by Dr. Phillip to make the following experiment. Having allowed

---

* " I made no reply to Mr. Brodie's observation, that he could discover only one point of difference between the experiment detailed by himself and his friends in the Quarterly Journal, and mine; because I could only repeat what I had said in the following number of that Journal. If Mr. Brodie had had occasion to repeat the experiment frequently, he would have seen, that all the circumstances I there mention are capable of influencing the result. It is needless, however, to insist on this, as he acknowledges that there was a deviation from my method which might have influenced the result: but I am assured that he will not obtain a correct result, till all the circumstances I mentioned, and particularly the proper strength of the galvanic power, as well as its mode of application, is attended to."

a full-grown rabbit to eat as much parsley as it chose, after a long fast, and shaved the hair off its stomach, I divided the eighth pair of nerves in its neck, and coated the lower part of the nerves with tin-foil, and bound a shilling over the stomach. It was then exposed to the galvanic influence, by connecting the tin-foil and shilling with the opposite ends of a galvanic trough of such power as to keep up a constant twitching in the fore-legs The difficulty of breathing, which always succeeds the division of the eighth pair of nerves, had come on before the galvanic apparatus was arranged. Upon the application of the galvanism, the breathing soon became free. When the power of the trough failed, or we intentionally discontinued the application of the galvanism, which we did repeatedly, the breathing always became oppressed; but was again rendered free on the re-application of the galvanism. This continued to be the case for twelve hours, after which the breathing could not be wholly relieved by the galvanism (inflammation of the lungs having supervened); and during the last hour of the animal's life the dyspnœa was very great. No attempts to vomit occurred in this animal, except once, when the galvanic influence had become very weak, ten or twelve hours after the division of the nerves; whereas, in every instance in which I have divided these nerves without the application of galvanism, and I have done so more than a dozen times in the rabbit, efforts to vomit always soon ensued.

"On examining the animal after death, the lungs were found highly inflamed. The contents of the stomach, instead of being such as above described, presented the appearance of those of a healthy stomach, in which digestion had been going on for many hours.

"I am ready to attest the truth of every part of the preceding statement in any way in which I may be called upon to do so.

(Signed) "CHARLES HASTINGS.

"*Worcester, February* 6, 1820."

"Three other medical men were present at this experiment, and particularly examined both the stomach and lungs; and expressed themselves perfectly satisfied with the result.

"C. H."

"Having, at the request of Dr. W. Philip, divided the eighth pair of nerves in the neck of two small dogs, which were allowed to eat as much lean raw mutton, cut into small pieces, as they chose, immediately before the experiment, after having fasted for many hours, I subjected one of them to the galvanic influence, by coating the lower parts of the divided nerves with tin-foil, and connecting it with one end of the galvanic trough; while the other end of the trough was connected with the region of the stomach, which, before the experiment, had been shaved, and a three shilling piece bound upon it. The power of the galvanism was such as to occasion a twitching of the fore-limbs during the whole experiment. The dog, which was not galvanized, was immediately seized with dyspnœa, and efforts to vomit. In the other, neither was observed in the slightest degree, at any period of the experiment, except when for a few seconds the galvanic influence was intentionally discontinued, during which the breathing became very laborious, again becoming free as soon as the galvanism was restored. This dog lived above two hours. The other dog was still alive, though extremely weak, at the end of four hours, at which time it was killed by a blow on the head.

"On examining the stomach of the galvanized dog, the mutton was found in a soft, half-dissolved state, all character of muscular fibre having disappeared. The lungs, on examination, were found perfectly healthy, but rather of a florid colour. In the stomach of the other dog the bits of mutton still retained their firmness, and on being cut into, displayed both the red colour and fibrous appearance of the muscle, which did not seem to be at all diminished. The lungs were found greatly congested, and collapsed very imperfectly, the surface being covered with patches of a dark red colour.

"The above experiment was made in presence of the house-surgeon and pupils of the Infirmary, who all examined the state of the stomach and lungs, and expressed themselves satisfied with the result. The accuracy of the above statement I am ready to attest in any way in which I may be required to do it.

(Signed) "JAMES P. SHEPPARD.

"*Worcester, February* 7, 1820."

"The following is the letter alluded to in the preceding:

"*Worcester, February* 6, 1820.

"SIR,—About ten days ago I wrote to you in consequence of some observations published in Dr. Cooke's work on nervous diseases. You will perceive that the occasion requires that my letter should be published. It will go to the press in a few days. I have requested a gentleman, on whose discretion I can depend, to deliver this to you, and to learn from you what reply, if any, you wish me to state, as having received from you, when I mentioned my having sent you the above letter.

"I have the honour to be,

"Sir,

"Your very obedient humble servant,

(Signed) "A. P. W. PHILIP.

"To B. C. Brodie, Esq."

"After waiting till the 10th for Mr. Brodie's reply to these letters, I addressed the following letter to that gentleman, with regret that the advanced period of the month did not allow me to wait a longer time for his reply than the 13th."

"*Worcester, February* 10, 1820.

"Sir,—I am sorry again to trouble you so soon; but as from the introduction to Dr. Cooke's work, and other reasons, I feel it necessary that the letters which I have addressed to you should be laid before the public, and am particularly anxious that your reply should appear in the form most agreeable to you; it is necessary that I should inform you, that my letters, for reasons with which I need not trouble you, must leave this for the press on the 14th instant, before I can hear from you by the mail of that day. As I have not heard from you in reply either to my letter of the 7th, or to that delivered to you by my uncle on that day, in which I mention the intended publication of my first letter, and beg to know what, if any, reply you wish me to make public, I am obliged to write to day, that you may not be called upon to reply by return of post; and I am very sorry, and beg to apologize, that circumstances oblige me to solicit so early a reply as I now do, which your numerous professional engagements may render inconvenient to you. If I hear from you, I shall of course follow the directions you give respecting the letter I have received from you. If not, I shall consider your silence as leaving me to the dictates of my own feelings. In that case I shall act as I should wish another to act towards me under the same circumstances, and give to the public, with my letters, your reply to my first letter; and I shall take care afterwards to make equally public any reply you may favour me with to my other letters, as far as you wish me to do so.

"I have the honour to remain,
"Sir,
"Your faithful humble servant,
(Signed) "A. P. W. PHILIP.

"To B. C. Brodie, Esq."

I received the following answer from Mr. Brodie on the thirteenth : —

*Saville Row, February* 12, 1820.

Sir, — I beg to acknowledge the receipt of your letters on the seventh and twelfth instant, and of Dr. Hastings and Mr. Sheppard's additions to the former.

In the experiments related in my last letter, the twitchings of the fore legs were nearly constant. There was no vomiting. It was not observed that the respiration was different from that of the other rabbit, which was not galvanized. The food in the stomach had the appearance of masticated parsley.

I shall be glad of the opportunity of seeing you make the experiment whenever you visit London. I undertake to promise that every facility shall be afforded you of doing so. I assure you it will give me much satisfaction to observe such results as you anticipate.

In closing this correspondence, I beg to remark, that you are in an error if you suppose that I laid the repetition of your experiment before the Royal Society with a view to influence any of its members against you, without your being able to vindicate yourself; I only joined two other gentlemen in repeating the experiment, at the request, and for the satisfaction of those with whom the management of the concerns of the Royal Society principally rests.

You are at perfect liberty to publish my letters; and I have the honour to be,

Sir,
Your obedient humble Servant,
(Signed) B. C. BRODIE

Dr. W. Philip, Worcester.

To this letter I returned the following answer : —

*Worcester, February* 14, 1820

"Sir, — I am favoured with your letter, and am much obliged by the readiness with which you offer me every facility in the repetition of my experiment. I shall have much pleasure in making it with you, and, from your answer to several of my queries, I have little doubt that we shall detect the cause of the difference of result. You say in reply to my last query, 'The food in the stomach had the appearance and odour of masticated parsley:' by this I understand that the parsley was, throughout the stomach, in the state here described. This state is very different from that in which the contents of the stomach were found in your first experiment; (see the Quarterly Journal for April last, page 164;) for which, as I observed in my reply to it, I could not account. You say in reply to my fourth query, that 'There was no vomiting' in the galvanized rabbit; that is, no efforts to vomit, as nothing is ever brought up after the division of the eighth pair of nerves. It appears from the observations in the 154th and 155th pages of my Inquiry, that we have reason to believe that no means will prevent the efforts to vomit after the division of these nerves but some degree of digestion going on in the food which lies in contact with the coats of the stomach. You observe in reply to my second query, that the twitchings of the fore legs were only 'nearly constant;' and, in your experiment, the galvanism was only continued for seven hours, while in the most decisive of my experiments on rabbits, it was continued for sixteen hours. In the result of my last galvanic experiment, in which the stomach was exposed only to a comparatively small degree of the galvanic influence, you will observe a striking resemblance to the result of your experiment. It is said in page 232 of my Inquiry, (second edition) 'The food was but little altered, retaining the colour, smell, and stringiness of the parsley. It was, however, sufficiently exposed to the galvanic influence to prevent the vomiting.' This perfectly corresponds with your account of the state of the contents of the stomach in your last experiment, but is very different from that given of them in the former. In my experiments, the metal was always applied to the lower part of the pit of the stomach. When it is applied near to the end of the sternum, hardly any part of the stomach is in the galvanic circle. You forget to answer my first query.

" It is a circumstance in this discussion, you will allow which particularly deserves notice, that the proofs on our side are all of a positive, yours, necessarily, of a negative kind. We only speak of what we could not have seen, had it not existed; you, of what you were not able to see, which many things besides what you suppose might have prevented your seeing. You now admit that in your first experiment there was a deviation from my method, which might have occasioned a difference of result, yet you were then quite as sure of the contrary in regard to that experiment, as you now are with regard to the present; for you say in the Quarterly Journal for April last, page 162, 'That the experiment might be repeated with the greater accuracy, the paper was put into these gentlemens' hands, *who implicitly followed the directions contained in it.*'

"If you will take the trouble to reperuse my letter, you will find that I do not even surmise that your motive in any thing you have done was such as you mention. I merely stated the facts and their necessary consequence: and now that you are sensible that the experiment was not a correct repetition of mine, you will regret, I am persuaded, that it was presented to the society.

I have the honour to be,
Sir,
Your faithful humble servant,
(Signed) A. P. W. PHILIP."

About a fortnight* after the date of the preceding letter, I addressed the following to Mr. Brodie: —

"*Worcester, March* 3, 1820.

"Sir, — As I have received no answer to my first query, relating to the manner in which the animal was prepared for your experiment, and this appears to me of considerable consequence in settling the point between us, I will thank you for your answer; and also to inform me, whether there was any appearance of inflammation in the stomach of either of the rabbits.

I have the honour to be,
Sir,
Your faithful humble servant,
A. P. W. PHILIP."

To B. C. Brodie, Esq.

The following was Mr. Brodie's answer, and the reply I made to it: —

* The Journals of last month having been published previous to the conclusion of the correspondence, the following letters are now printed for the first time.

"*Saville Row, March* 5, 1820.

"Sir,—Both animals in my last experiment were fed after a fast of about sixteen hours. I did not observe any appearance of inflammation in the stomach of either of them, but my attention was not particularly directed to this point.

I have the honour to be,
Sir,
Your obedient servant,
B. C. BRODIE."

Dr. Wilson Philip.

*Worcester, March* 9, 1820.

"Sir,—I yesterday received your reply to my last letter, from which it appears, that the animals were properly prepared for the experiment. Your answer to my question respecting the appearance of inflammation, greatly surprises me, as by referring to my Inquiry, you will see, that I have from the first, regarded inflammation of the stomach as the criterion that the proper power of galvanism had been employed. In the 216th page of the first edition, it is said, 'We could never succeed;' that is, in the newly dead animal, 'in producing the slightest appearance of inflammation, either in the stomach or bowels, *an effect which uniformly attends digestion, supported by galvanism.*' And, again, page 135, 'The thoracic viscera, in short, were rather in a state of high inflammation, *an effect always produced by a considerable galvanic power in the part to which it is directed.*' I, therefore, suspected, that in repeating my experiment, your attention would have been particularly directed to this circumstance. It is impossible, however, to see a stomach which has for a sufficient length of time been exposed to such a power of galvanism as I have found necessary to produce digestion, without being struck with its inflamed appearance.*

"I mentioned, in my letter of the 14th ult. such reasons as assured me that the stomach, in your last experiment, had not been sufficiently exposed to the galvanic influence. What you now state, leaves no doubt on this head. As there was little or no inflammation excited in the stomach,

* "The inflammation of the organ, to which the galvanism is chiefly directed, is easily accounted for, as it is not to be supposed that we can, by the artificial application of galvanism, direct it in the proper quantity to the proper parts, in such a way, as not to supply more to some, and less to others, than nature supplies."

it is *certain*, that the galvanic power which you employed was either too weak, or not sufficiently directed to the stomach. We need look no farther for the difference of result between your experiment and mine. Thus, it appears, that both your repetitions of my experiment, leave the subject in precisely the same predicament, as if neither of them had been made, with this exception, that the efforts to vomit being prevented by so small a power of galvanism, affords a striking, because an unintentional, confirmation of the result I obtained.

"In case the experiment should again be repeated, I think it necessary to call your attention to the following circumstances, which are not less essential than the degree of the galvanism and its mode of application. Although your experiments stand in opposition only to my shorter experiment on rabbits, (for as to that in which the galvanism was continued for sixteen hours, no attempt has been made to repeat it) yet you adopt the rule which, according to my method, necessarily produces an experiment of long continuance; for your object is to employ no stronger power of galvanism than is requisite to produce a twitching in the fore legs. In my shorter experiment, which lasted six hours, the power of the galvanism was much beyond this degree, producing *general contractions* of the muscles, (p. 224, second edition,) and proving fatal to the animal in the above short space of time. I found it more conclusive to employ a comparatively weak power of galvanism, that the animal might be longer exposed to it; but you employed this weak power, and yet, by killing the animal at the end of five or seven hours, defeated my object in having recourse to it. You wholly deviated from my principle, when you killed the animal by any other means than the galvanism. You will perceive, from all that I have had occasion to say, how far from correct is the observation in your first letter, that you could discover but one point of difference between your experiment and mine.

"It is also proper to remark, that the old galvanic trough, which I observe in my Treatise, I always used, is preferable in such experiments to the improved pile, because, in the former, the number of plates is greater in proportion to the surface; and I have found, that it is on the intensity, not on the quantity of the electric power, that the above effects on the animal body depend. See some observations on this subject which I transmitted to Dr. Thomson, and which appeared in the eleventh volume of the Annals of Philosophy, page 117.*

I have the honour to be,

Sir,

Your faithful humble servant,

A. P. W. PHILIP."

"To B. C. Brodie, Esq."

"Having occasion to address the public on the subject of the preceding letters, I beg leave to make some further observations connected with it. I do not feel myself called upon to reply to various observations which have been publicly made on the opinions maintained in my Inquiry into the Laws of the Vital Functions. I feel no anxiety in leaving them to the judgment of those who are versed in the subjects to which they relate; but there are two instances in which my meaning has been so unaccountably misunderstood, and that in works which are in the hands of many members of our profession, that without some explanation on my part, I must appear to maintain opinions which I believe to be wholly unfounded.

"The first of these works which I shall mention is entitled, *Additional Experiments on the Arteries of Warm-blooded Animals, &c.* by Charles Henry Parry, M. D. F. R. S. &c. In the Inquiry above mentioned, I had occasion to reply to some observations of Dr. Parry (father of Dr. C. H. Parry), in his *Elements of Pathology and Therapeutics,* on the opinion which I had maintained respecting the nature of inflammation. The latter gentleman has given an answer to this reply in the above Treatise. It is with reluctance that I notice his answer, because I am wholly at a loss to account for his, I may almost say, uniform misconception of my meaning.

"In the 160th page he begins his answer by observing, 'Dr. Wilson Philip, taking up the opinion of Bichat and

* "Although I have in my first letter replied to an experiment of Mr. Brodie's on the frog, I think it proper to observe in concluding this correspondence, that had the wound healed perfectly in this experiment, it could not have been adduced as an argument against inferences from experiments on the warm blooded animal; for the functions of the nervous system in warm and cold blooded animals differ so much, that the frog will perform motions of volition, and even sit in its usual posture for several days after the head is removed. When we descend lower in the scale, we find animals capable of regenerating whole members, and, at length, some, the different parts of which, when they are cut into pieces, live as distinct animals."

others, has, indeed, attempted a direct proof from experiment, in favour of the exclusive influence of the capillaries in carrying on the circulation.' If Dr. C. H. Parry can point out any passage in my Treatise in which such an opinion is expressed, I shall be much more surprised than he can possibly be, that any one should maintain a position so extravagant. Many quotations from my Treatise follow, the meaning of all of which he appears to me to misapprehend. I would ask Dr. C. H. Parry, where I have maintained that the larger arteries do not possess 'an equal and proportional share of power (page 161) with the capillaries?' Where have I made any inference from the irregular motion of the blood, observed under certain circumstances in the capillaries, respecting the cause of circulation? (page 163.) Where have I maintained that 'no influence can be exerted through them,' namely, the heart and large vessels, 'by stimuli on the capillaries?' (p. 168.) With respect to the quotation in page 165, if Dr. C. H. Parry will take the trouble to re-peruse it, he will find that I speak of the result of Dr. Parry's experiments, not of any thing which 'Dr. Parry has himself asserted.' Similar observations apply to other passages.

"I must beg the reader's patience, while I say a few words in reply to the following observations of Dr. C. H. Parry, on the quotation which he gives from my Treatise, in page 175. He observes: 'Dr. W. Philip continues, the truth of this inference appears, indeed, from direct experiments; for from those made with a view to ascertain the state of the vessels in an inflamed part, it clearly appears, not only that the velocity of every part of the blood was lessened, which Dr. Parry admits may be the case, supposing the lessened momentum arising from this cause more than compensated by the increased quantity of blood; but that the general momentum of the blood also, in the inflamed part, was lessened, because the blood was observed to move more and more slowly, till, in the most inflamed part, it ceased to move altogether.'

"Dr. C. H. Parry's first observation on the above passage, is—'This sentence is certainly not intelligible.' It would, possibly, have been more correct, had he said, 'not intelligible to me.' 'In the first place,' Dr. C. H. Parry proceeds, 'Dr. Parry has no admission with regard to diminished velocity, which is entirely an assumption of the author, but simply as to the rate of momentum.' Dr. Parry observes, in his *Elements of Pathology and Therapeutics*,—'Neither will this conclusion be invalidated, were it even proved, according to the opinion of Dr. Wilson, that the velocity of the blood in the vessels of an inflamed part is diminished, unless it be also proved, that the velocity is diminished in a greater proportion than the quantity is increased.' This passage I quoted, and only argued on it to shew, that the velocity is diminished more than the quantity is increased.

"In the second place, Dr. C. H. Parry proceeds—'When we consider that the momentum is the product of the quantity and velocity, it is not possible to suppose 'a lessened momentum more than compensated by an increased quantity of blood.' 'If, in this passage, the term velocity had been substituted for momentum, there would have been some appearance of meaning.' The passage here given as a quotation from my Treatise, is, indeed, nonsense; but it is Dr. C. H. Parry's, not mine. The reader will observe, from the quotation in the last paragraph but one, that the passage in my Treatise stands thus—'Supposing the lessened momentum arising from this cause,' namely, the lessened velocity, 'more than compensated by the increased quantity of blood.' This, to most men, I believe, would be perfectly intelligible.

"In the 174th page, Dr. C. H. Parry also uses very strong expressions, under similar circumstances. He observes of me—'In combating Dr. Parry's opinions on these subjects, he thus expresses himself: 'Does it not seem a necessary inference, that the blood is moved in the capillaries by the power of these vessels themselves; and, consequently, that if they are debilitated, the momentum of the whole part, as well as its velocity, must be less than in health?' Now, though this is tautological, and superfluous as relating to a case where the quantity of matter is allowed to be increased, because there is no instance in which the momentum is diminished, where the quantity is increased, without such a diminution of velocity,' &c. If Dr. C. H. Parry will take the trouble to refer to my Treatise, he will find that he has again misquoted it. He, indeed, makes the sentence such, as deserves even more than the severity with which he treats it.

"I shall trouble the reader with but one more passage from Dr. C. H. Parry's Treatise, (page 177.) 'With regard to some of Dr. Wilson Philip's experiments, I must confess that I have repeated them with very different results. To avoid the cruel, because sometimes uncertain, process of crushing the brain with the blow of a hammer, and to remove altogether the injurious effects of the violent motion which it may occasion, I have enclosed the whole head in a pair of wide and flat-mouthed pincers, with large handles, forming a powerful lever. The whole head was thus, without the least disturbance, or chance of an imperfect effect, crushed in the smallest possible space of time. The result of this sudden and certain death has not been a suspension of the capillary action, but its increase.' Dr. C. H. Parry must be aware, that when the brain is crushed by the blow of a hammer, which can never be uncertain if the hammer is very large, its destruction is instantaneous. It occupies but a small fraction of the time which must be employed in crushing it by the means which Dr. C. H. Parry employed. His remark, therefore, that it was crushed in the smallest possible space of time, cannot be admitted. The effect in his experiment was the same as that which I have mentioned as ensuing when the brain is imperfectly crushed.—See my Inquiry, page 94, second edition. If Dr. C. H. Parry will take the trouble to look into it, he will see how much the result of such experiments depends on the suddenness with which the brain is crushed. Is it fair, in repeating physiological experiments, to deviate from the method of the original experimenter in the most essential respects, and then adduce the necessary difference of result, as a proof of his inaccuracy?

"I regret that I have been obliged to reply to a son of Dr. Parry in the way in which I have now done. Dr. C. H. Parry cannot find a better example than that of such a father. He will see in his writings nothing that had not been duly considered; and his replies, to those who dissented from him, are remarkable for the modesty of a philosopher, and the language of a scholar and a gentleman.

"The other work above referred to, is the London Medical and Physical Journal. In a Proemium to the 43d volume of this work, by Mr. Hutchinson, just published, in which this gentleman has taken upon himself the duty of instructing the public, respecting 'the progress of medicine, and its auxiliary sciences,' there are many observations relating to my Inquiry into the Laws of the Vital Functions. In the 20th page he observes of me—'He also asserts, that he has refuted the notions of Bichat, respecting the functions of the ganglionic system of nerves, although the positive argument of the life and growth of the fœtus, without brain and spinal marrow, may alone be considered a sufficient refutation of the negative arguments, formed, for the most part, on loose and remote analogies, which Dr. Philip advances against the doctrines of Bichat.' What is meant by my assertion, that I have refuted the notions of Bichat respecting the ganglian system, and the loose and remote analogies, I know not; unless they allude to my referring the reader to certain experiments, detailed in my Inquiry, the results of which are inconsistent with his opinions on that subject. In a note referred to, in what Mr. Hutchinson says respecting the fœtus without brain and spinal marrow, it is observed:—' Dr. Philip does not even notice this fact, although there are instances of it recorded by Morgagni,' &c. Now, I have not only mentioned this fact repeatedly, but have entered into a disquisition of some length respecting it, in both editions of my Inquiry*; and in the second edition, described a case of this kind, which I had an opportunity of examining†.

" Mr. Hutchinson continues:—' The only important argument brought against the doctrine of Bichat, is, that the functions he stated to depend on the influence of the ganglionic system of nerves, cease on the destruction of the brain and spinal marrow,' (page 21.) This gentleman here passes unnoticed, various other parts of my Treatise; all the experiments, for example, which I have detailed for the purpose of proving that the organs, which are under the ganglian system, are affected by stimuli applied to the brain and spinal marrow. He does not seem aware that I had ever made such experiments.

" A little lower, in the same paragraph, he observes:—' The experiments of Dr. Philip shew similar results; but there is one peculiar observation of his, that is very interesting; which is, that when the action of the heart has been much disturbed by the destruction of a certain portion of the spinal marrow, by waiting a little time, that action would resume its former regularity, and then another portion of the spinal marrow might be destroyed without stopping it, and so on repeatedly.' At the end of this sentence there is a reference to my Inquiry, and even to the particular experiments; experiments which I never made, as Mr. Hutchinson will find, if he ever takes the trouble to read my Inquiry. All this would be quite amusing, were not so gross a violation of the implied compact

* " First edition, page 61, pages 240, 241, 242, 243. Second edition, page 61, pages 250, 251, 252, 253.

† " Second edition, page 62.

between a reviewer and the public, too grave a subject for merriment.

"As Mr. Hutchinson appears to be wholly unacquainted with the contents of my Inquiry, it is not surprising that in page 22 he should argue against my supposed opinion, that the existence of some part of the spinal marrow is necessary to the action of the heart; although I have detailed many experiments, in the very beginning of that Inquiry, for the purpose of proving, that the power of the heart is wholly independent of every part of the spinal marrow, its action not being in the least influenced by the total removal of that organ. In the next paragraph he ascribes to me the opinion, that the ganglian system retains the nervous influence, 'like a sort of reservoir;' whereas, I have made many experiments, and detailed many observations, expressly with a view to prove, that the ganglian system is not a reservoir of nervous influence, but a mere channel through which it flows. But the reader has seen enough of Mr. Hutchinson's suppositions. I do not mean to say, that he is intentionally incorrect; but the effect, as far as relates to the public, is the same as if he were so; and such unaccountable carelessness in a man, who professes to judge others, and direct public opinion, is little less culpable than intentional error.

"Where there is such incorrectness in the statements, we expect to find equal inaccuracy in the reasoning. Mr. Hutchinson's reasoning does not disappoint this expectation."

In answer to Mr. Hutchinson's remarks, in the last Number of the Medical and Physical Journal, on the foregoing observations, I must say, that he and I appear to attach a very different degree of importance to the misstatements of a Reviewer. Much as he complains of my censure, it has produced little amendment; for, in the very beginning of his reply, he calls M. Legallois "the executor of those experiments which most clearly prove," that the action of the heart continues after the destruction of the spinal marrow. Now, all who are acquainted with the work of M. Legallois know, that his chief object was to prove, and that all his experiments relating to the subject tend to prove, that the action of the heart does *not* continue after the destruction of the spinal marrow. Many of my experiments were made for the express purpose of refuting this opinion of M. Legallois.—He calls me "the repeater of the *greater part* of Legallois's Experiments."

In the whole of my Treatise I relate the repetition of but *one* of this author's experiments, and that in a cursory way, and for the purpose of comparing it with one of my own.

Mr. Hutchinson quotes from my Treatise observations which apply to *the nerves in general,* as a proof that I consider *the ganglian system* a reservoir of nervous influence! According to this new explanation, every nerve is a reservoir of nervous influence. He forgets that his former remarks applied to the ganglian system exclusively.

I do not wish to proceed farther; but let Mr. Hutchinson *allow himself time* to compare his Reply with my Treatise, and with the observations which his account of it has drawn from me, and he will have no cause to charge me with exposing *slight* inaccuracies. The hurry of Mr. Hutchinson's feelings prevents his seeing, that I am not guilty of the harshness of which he accuses me. "This is an error," he observes, "I confess it with regret; but the branding it with the epithet of a gross violation of the implied compact between a Reviewer and the Public," &c. If he recur to my observation he will find, that it was applied not to one, but many errors, which I had enumerated, as appears from the first words of the sentence, "All this."

When I censured Mr. Hutchinson's reasoning, I ought perhaps to have given an instance of what I censured. On farther reflection, he will, I think, acknowledge, that what he says of the nervous influence, towards the conclusion of his remarks on my Treatise, can only be correct on the supposition, that we can, after the removal of the brain, excite to its usual function a secreting surface, as we can excite a muscle, by irritating it with the scalpel. That this is not the case, Mr. Hutchinson, I suppose, will admit.

*Additional*

