# Errata in Dr. Philip's Paper, in Last Number

**Published:** 1820-06-01

**Authors:** 


### *Errata in Dr. Philip's Paper, in last Number.*

Page 659, line 30, before "that" insert "*is*;"

680, 6, for "mentioned," read "*mention*."

683, line last but one besides the note, for "was," read "*were*."

684, line 28, for "suspected," read "*expected*."

